# Advances in genetics: widening our understanding of prostate cancer

**DOI:** 10.12688/f1000research.8019.1

**Published:** 2016-06-27

**Authors:** Angela C. Pine, Flavia F. Fioretti, Greg N. Brooke, Charlotte L. Bevan

**Affiliations:** 1 Molecular Oncology, School of Biological Sciences, University of Essex, Colchester, Essex, UK; 2 Androgen Signalling Laboratory, Division of Cancer, Department of Surgery and Cancer, Imperial Centre for Translational & Experimental Medicine, Imperial College London, London, UK

**Keywords:** Prostate Cancer, Whole Exome Sequencing, WES, Personalised Medicine, Androgen, Transcriptome, Metastasis, Cancer progression, DNA damage repair

## Abstract

Prostate cancer is a leading cause of cancer-related death in Western men. Our understanding of the genetic alterations associated with disease predisposition, development, progression, and therapy response is rapidly improving, at least in part, owing to the development of next-generation sequencing technologies. Large advances have been made in our understanding of the genetics of prostate cancer through the application of whole-exome sequencing, and this review summarises recent advances in this field and discusses how exome sequencing could be used clinically to promote personalised medicine for prostate cancer patients.

## Introduction

Prostate cancer (PCa) is the most common cancer among men in the UK, with over 40,000 cases diagnosed every year ^1^. More than 10,000 men die from the disease in the UK per annum, making it the second most common cause of cancer-related death behind only lung cancer. Similarly, in the USA, PCa accounts for just over a quarter of all cancer diagnoses in men, with 220,800 diagnoses and 27,540 deaths from PCa predicted in 2015 ^2^. Statistically significant risk factors associated with PCa include ethnicity, family history of the disease, and age ^3^, with over 75% of all PCa cases diagnosed in men over 65 years of age ^1^. Other factors such as cigarette smoking history, lower physical activity, higher body mass index, and height are associated with increased risk of fatal disease ^3, 4^.

PCa growth is dependent upon the androgen receptor (AR), a ligand-dependent transcription factor. In response to androgen binding (the major AR ligand in prostate is dihydrotestosterone), the AR regulates the expression of target genes/proteins important in PCa growth (e.g. 5– 7). The treatment given to patients with PCa is dependent on the grade and stage of the disease ^8^. PCa that is contained within the prostate capsule can be removed via surgery to remove the prostate or treated using radiotherapy ^9^. Given the potential side effects and the fact that low-grade tumours often grow slowly and may not become clinically significant, many patients, especially if they are older, tend to be monitored rather than treated (termed “active surveillance”).

Since androgenic hormones drive prostate tumour growth, therapies that target the androgen signalling pathway are commonly used for tumours that have spread outside the capsule ^10^. These initial hormonal therapies fall into two categories ^11, 12^. The first blocks the gonadal production of androgens by pituitary downregulation. This can be achieved using luteinising hormone releasing hormone (LHRH) analogues. They cause an initial spike in androgen levels; however, within 2 weeks, castrate levels of circulating testosterone are achieved due to hyperstimulation of the hypothalamo-pituitary axis. In contrast, anti-androgen therapies (e.g. bicalutamide and enzalutamide) do not reduce androgen levels *per se* but act directly on the AR; their binding to it results in the AR adopting an inactive conformation with subsequent inhibition of downstream events. The two approaches may be used sequentially with a switch in treatment when the first fails, or simultaneously in complete androgen blockade. However, although such hormone therapy is initially successful in the majority of patients, it invariably eventually fails, with tumours becoming unresponsive to therapy within 1–3 years and tumours progressing to the aggressive stage termed castration-resistant PCa (CRPC). Although in recent years several new therapies have been developed with some licensed for use in CRPC ^13, 14^, there remain few effective options and the mean survival period for patients with CRPC in 2012 was just 13.5 months ^15^. There is therefore a great need to identify therapeutic strategies to prevent/treat CRPC but also to develop the means and biomarkers to stratify patients for optimal therapy, and the genetic information from the studies described below is a major step in this process.

## Whole-exome sequencing

The human genome consists of approximately 3 × 10 ^9^ base pairs. Only around 1% of these (3 × 10 ^7^ base pairs) is believed to represent coding sequence, but it is estimated that 85% of disease-causing mutations are located in these coding regions of the genome – collectively termed the exome ^16, 17^. Hence, most studies to date have concentrated on characterizing the exome, initially indirectly, through microarray expression analyses, and now owing to advances in DNA sequencing technologies by whole-exome sequencing (WES). WES, on which this review focuses, has led the way in uncovering mutations in coding regions responsible for many diseases and in practical and economic terms is, for many, more feasible than whole-genome sequencing (WGS), although it will not identify changes in non-coding regions of the genome (see later) ^18^. Hence, availability of financial resources as well as project-specific requirements (such as the ability to detect splice variants, gene fusions, and non-coding transcripts) are likely to influence decisions on whether to employ WGS, transcriptomic sequencing, WES, or other forms of targeted re-sequencing (such as deep sequencing of targeted gene panels).

In WES, DNA sequences are isolated only from exons, and data analysis compares the patient sequence to that of a reference exome aligning all of the captured exons. The variants found are compared to a control population database containing non-disease-causing variants. After the common variants are filtered out, the data can be compared to the exomes of unaffected individuals or normal tissue to identify disease-associated variants ^19^. The first exome sequencing study to be reported was performed by Ng *et al*. ^20^, in which the exomes of 12 individuals with Freeman–Sheldon syndrome (FSS), a rare dominantly inherited disorder, were sequenced. In agreement with previous reports ^21^, the study demonstrated that mutations of embryonic myosin heavy chain ( *MYH3*) were present in patients with the disease. Since this study, the amount of literature published using WES technology, and the range of diseases, has been increasing exponentially. Furthermore, the successful diagnostic rate of rare disease using this technology has now reached 25% ^22^, with many clinical laboratories offering WES as a cost-effective means of clinical testing and diagnosis ^23^.

WES is preferably performed on DNA obtained from fresh or frozen patient samples. The issue of obtaining fresh PCa tissue for genome analysis was recently addressed by Menon and colleagues, who used WES to compare fresh samples and formalin-fixed paraffin-embedded (FFPE) material from the same patient ^24^. The study demonstrated a high degree of overlap in single nucleotide variations in both types of samples, suggesting that FFPE material is a viable option for such studies. This supports previous work by Schweiger *et al*. and Kerick *et al*. ^25, 26^ and is a major consideration for PCa research, since it supports the use of archival FFPE biopsy samples for WES.

The availability of samples from advanced metastatic PCa is historically limited to biopsies taken from the primary tumour; analysis of tumours to, for example, identify mechanisms of therapy resistance has therefore been hampered by lack of material. This situation is improving, with metastatic samples taken post-mortem in systematic approaches such as the expanding “warm autopsy” program developed at the University of Michigan by Rubin, Pienta, and colleagues ^27^ and recently the move to biopsy metastatic tumours from living patients, as exemplified in the landmark paper from Robinson *et al.* using both WES and transcriptomic sequencing to characterize genetic lesions in 150 metastatic CRPC patients, both at bone and soft tissue metastatic sites ^28^. Also, in recent years, there has been a move towards non-invasive sampling (liquid biopsies such as plasma, serum, urine, and semen) to obtain samples for WES analysis and biomarkers in general. For example, Mutaza and colleagues performed WES on circulating cell-free tumour DNA (ccfDNA) obtained from the plasma of patients ^29^. The study identified a number of mutations associated with drug resistance, e.g. an activating mutation in phosphatidylinositol-4,5-bisphosphate 3-kinase (PIK3CA) found following paclitaxel treatment. The ability to use liquid biopsies in the prostate field will circumvent issues of material availability and is an important development in the field of WES use for personalised medicine.

## Identification of gene alterations associated with prostate cancer susceptibility

Since familial PCa was first described in the 1950s ^30^, there has been a strong search for hereditary mutations linked with the occurrence of the disease. For example, genome-wide association studies (GWAS) and single nucleotide polymorphism (SNP) arrays have identified more than 100 PCa susceptibility loci ^31, 32^. The majority of these SNPs are located in non-coding regions, and bioinformatic approaches have been used to identify candidate genes affected by these variants ^33^.

WES has also been used to identify genetic variants in coding regions that correlate with PCa predisposition. The G84E mutation in the homeobox transcription factor HOXB13 is an example of such a SNP strongly associated with early onset familial PCa, confirmed through parallel targeted sequencing of germline DNA from 94 unrelated PCa patients and their families ^34– 36^. Similarly, both BRCA1 and BRCA2 have been linked to PCa predisposition, although no specific SNP has been identified, rather a variety of genetic alterations (e.g. protein-truncating mutations, in-frame deletions, and missense variants) in the two genes that cause loss of protein function ^37, 38^. The IMPACT study showed, in fact, that targeting prostate-specific antigen (PSA) screening to men bearing BRCA mutations identified a higher proportion with PCa and that BRCA mutation-positive men are more likely to have an aggressive form of the disease ^39^.

Rand *et al*. ^40^ compared the exomes of 2165 PCa cases and 2034 controls of African ancestry with the aim of identifying protein-coding variations that affect disease risk in this population. Among the significant associations identified were mutations in Secreted Protein Acidic and Rich in Cysteine-Like 1 (SPARCL1) and Protein Tyrosine Phosphatase, Receptor Type, R (PTPRR). SPARCL1 has been shown to have tumour suppressor activity ^41^, and the alanine to aspartic acid substitution at amino acid 49 is associated with reduced PCa risk (odds ratio [OR] = 0.78, p = 1.8 × 10 ^-6^). In contrast, the substitution identified in PTPRR (Val239Ile), an AR target gene and regulator of the RAS/ERK1/2 pathway ^42^, was associated with increased risk (OR = 1.62, p = 2.5 × 10 ^-5^). In another WES study of individuals from families with three or more affected individuals, it is notable that several of the changes associated with PCa were in genes linked to DNA damage repair, including three poly(adenosine diphosphate [ADP]-ribose) polymerase (PARP) genes ^43^; this is especially interesting given recent reports of clinical benefit conferred by PARP inhibitors in PCa patients with defects in DNA-repair genes ^44^.

## Gene alterations associated with prostate cancer development/progression

A number of genetic alterations have been correlated with PCa development and progression. Perhaps unsurprisingly, the recent comprehensive profiling study of metastatic CRPC using WES and transcriptomic sequencing found mutations in genes in the AR signalling pathway in over 71% of cases: the majority were in the *AR* gene itself ^28^. Since Taplin *et al*.’s original report of AR mutation and Visakorpi *et al*.’s report of AR amplification in advanced PCa, many more have been published ^45, 46^. These alterations are rare in early stages of the disease and appear in response to selective pressure resulting from the hormone therapies administered, allowing the receptor to continue to drive growth in CRPC ^10, 47– 53^. There are many reports of missense mutations leading to amino acid substitutions, usually within the ligand-binding region, which broaden ligand specificity to allow activation by, for example, adrenal androgens, glucocorticoids, and even therapeutic antiandrogens ^54– 58^. More recently, constitutively active AR splice variants have been identified, which circumvent the requirement for ligand ^59, 60^. To date, at least 20 such variants have been described, all of which lack the ligand-binding domain, are nuclear in the absence of ligand, and have been reported to have constitutive ligand-independent activity, although some studies suggest they still require the presence of the full-length receptor for activity ^60– 64^. As well as alterations of AR, mutations of other components of the AR signalling pathway have been found to correlate with disease progression. In their landmark study, Grasso *et al*. compared the exomes of 50 heavily treated metastatic CRPC tumours with 11 high-grade treatment-naïve non-metastatic tumours ^65^, and a number of genes encoding proteins that interact with, and/or regulate the activity of, the AR were found to be altered. For example, mixed-lineage leukemia protein 2/histone-lysine N-methyltransferase 2D (MLL2/KMT2D), a histone-modifying enzyme that interacts with the AR, was mutated in 8.6% of cancers. Alterations were also found in FOXA1 (3.4% of cases), a pioneer factor that de-compacts DNA allowing genomic access of nuclear receptors, including the AR ^66^. The majority of FOXA1 mutations and indels identified were in the carboxy-terminal transactivation domain, and functional assays demonstrated that these alterations enhanced tumour growth. In support of other studies, AR mutations and an increase in copy number were also identified in the majority of patients with advanced disease ^65^. In addition, the recent Robinson *et al*. paper highlighted that 71.3% of metastatic CRPC tumours carried AR pathway mutations, the majority in the AR itself but others in e.g. AR cofactors (NCoR1/2) and, again, the pioneer factor FOXA1 ^28^.

The gene often cited as most frequently mutated in primary PCa, encoding Speckle-type POZ protein (SPOP), is mutated in over 10% of primary prostate tumours ^67^. Mutations in this gene appear early in development and impact the ability of the cell to repair DNA damage, leading to genomic instability ^68^. Other targets of SPOP include the AR and the ERG oncogene, confirming the importance of this gene in driving tumour progression ^69^.

Phosphatase and Tensin homolog (PTEN) is found mutated at a similar frequency in primary PCa (10%), while PTEN deletion occurs in up to 70% of surgically treated cancers and over 60% of metastatic prostate tumours ^70– 73^. PTEN mutations or copy loss leads to increased PI3K/Akt signalling, which translates into cell survival and proliferation, e.g. through ligand-independent activation of the AR signaling pathway ^74^. It has been hypothesised that PTEN deletion creates genomic instability that then facilitates other alterations, e.g. the TMPRSS-ERG fusion commonly found in PCa ^75, 76^. The TMPRSS-ERG gene originates from fusion between the *TMPRSS2* promoter, which is androgen responsive, and the *ERG* gene ^77^. ERG is a member of the ETS family of transcription factors, which has roles in numerous processes including cell proliferation, apoptosis, differentiation, angiogenesis, and invasiveness. This gene fusion causes the oncogene ERG to be under the control of the androgen inducible TMPRSS2 promoter, which appears to have a subsequent bearing upon tumour progression ^78^. Although ERG is the most common fusion partner, other ETS genes (notably ETV1 and ETV5) can be fused to the TMPRS22 promoter in prostate tumours, and also mutations in ETS gene family members have been identified in tumours, prompting speculation that some of these may have tumour suppressive function ^65, 79^.

Loss of function of tumour suppressors is also a common event in PCa development and progression, and those frequently lost are p53, retinoblastoma (Rb), and NK3 transcription factor related, locus 1 (NKX3.1) ^80– 82^. Dysregulation of the tumour suppressor p53 is rarer in PCa than other tumour types, at around 5–10% in primary tumours, but increases to around 50% in metastatic CRPC ^28, 67, 83^. A recent study, using candidate gene exome sequencing, suggested that patients with dominant negative p53 mutations had the worst outcome, and this feature by itself has independent prognostic relevance for patients ^84^. NKX3.1 is an androgen-regulated gene known to regulate prostate organogenesis during embryogenesis and is expressed throughout adult life, where it regulates ductal function and secretion ^85^. Complete loss of NKX3.1 expression is evident in 5% of benign prostatic hyperplasias, 20% of high-grade prostatic intraepithelial neoplasias, 34% of hormone-refractory PCa, and 78% of metastases, supporting a role in disease progression ^65, 82^. In contrast to p53 and Rb, NKX3.1 tumour suppressor activity is restricted to the prostate and its loss of activity is usually due to absent protein expression rather than inactivating mutations ^86^.

**Figure 1.**
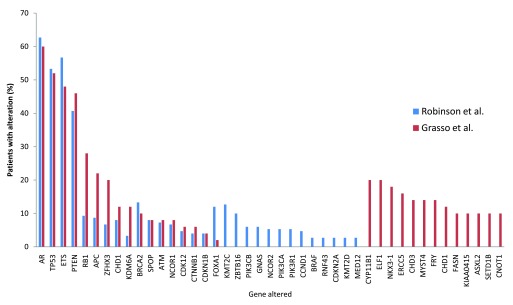
Common genetic alterations associated with advanced metastatic prostate cancer identified in two major whole-exome sequencing studies. The top 30 genetic alterations found in each of the studies by Robinson *et al*. and Grasso *et al*. ^28, 65^ were compared.

When comparing WES studies, some common alterations are evident. Taking the top 30 hits each from two recent comparable exome studies of advanced metastatic PCa, 17 genes were found to be altered in both studies ^28, 65^ ( Figure 1). For example, alterations in *AR*, *TP53*, *ETS* fusion, *PTEN*, and *RB1* were common to both studies. However, a number of other alterations were found in only one study, some with a relatively high incidence rate (e.g. Grasso *et al*. found *CYP11B1* to be altered in 20% of patients ^65^). The discrepancies between studies are likely to be resolved as the number of tumours sequenced increases but are also likely to represent the significant heterogeneity associated with PCa. Inevitably, key driver mutations will be found in multiple studies. Further comparison with other larger sequencing studies such as the 100,000 human genome project ^87^ will aid in the identification of the key/driver mutations and variants important in PCa initiation or progression, as these will be enriched in PCa compared to other diseases and the general population.

## The use of whole-exome sequencing for personalised medicine

There is an urgent need to stratify patients according to which therapeutic is likely to be most effective, to increase drug efficacy, and to reduce over-treatment and unnecessary side effects. WES holds great promise in this regard and has already been demonstrated to be a useful tool in terms of determining the cause of resistance to therapeutics or indeed why certain unconventional treatments may benefit patients with a particular cancer for which they would not normally be given. An example of this was an unexpected finding in a study conducted by Beltran and colleagues ^88^, which used WES to analyse the exomes of 97 patients with a range of metastatic cancers. One of the tumours analysed was from a PCa patient found to have an exceptional response to cisplatin treatment. Exome sequencing identified that the DNA repair protein FANCA had reduced expression and activity as a result of somatic hemizygous deletion and a partial loss of function as a result of a germline missense mutation in the second allele; subsequent assays demonstrated that loss of FANCA function was associated with platinum hypersensitivity, thus providing a rationale for the patient’s clinical response to an unconventional treatment. The widespread application of prospective WES in personalised medicine was also demonstrated in this study, since the authors were able to identify therapeutics (approved or in development), for 94% of the patients, expected to be effective given the exome profile generated ^88^. Robinson *et al*. reported a similar rate of “actionable” mutations in their study of metastatic CRPC, i.e. mutations on the basis of which informed treatment advice could be offered, including BRCA or ATM mutations that indicate use of PARP inhibitors ^28^. To date, each exome or transcriptome sequencing study of the prostate identifies a number of mutations unique to that study, while also a notable number of common mutations and/or affected genes. This provides both evidence for the key pathways and driver mutations in PCa and potential information leading to the effective application of a wide range of therapeutics.

## Concluding remarks

The launch of the International Cancer Genome Consortium ^89^ ( https://icgc.org) in 2008 paved the way for genome studies on over 50 cancer types and through the use of sequencing approaches, including WES, has significantly improved our understanding of the genomic, transcriptomic, and epigenomic changes associated with different tumour types. Repositories such as this are providing a valuable resource for researchers in the field. In comparison to complete genome sequencing, WES provides information only about alterations in the coding sequence; however, it is cost effective and the reduced data analysis associated with WES means that it is likely to continue to be a valuable tool for PCa research. It also holds great promise in the clinic, having the potential to assist and inform personalised medicine for men with the disease. Undoubtedly in the future, as transcriptomic approaches become more widely used, they will lead to similar advances relating to the non-coding portion of the genome; microRNAs, long non-coding RNAs, other non-coding RNAs, and epigenetic changes are also likely to yield markers for tumour classification as well as actionable mutations. Despite the seemingly unlimited potential of WES, the frequent lack of knowledge of the functional consequences of such gene alterations is an issue that requires addressing in subsequent functional studies. These will be invaluable in characterising the phenotypic consequences of these gene alterations and are likely to yield findings that can be exploited for therapeutic gain.
